# Gene Ontology annotation of sequence-specific DNA binding transcription factors: setting the stage for a large-scale curation effort

**DOI:** 10.1093/database/bat062

**Published:** 2013-08-27

**Authors:** Sushil Tripathi, Karen R. Christie, Rama Balakrishnan, Rachael Huntley, David P. Hill, Liv Thommesen, Judith A. Blake, Martin Kuiper, Astrid Lægreid

**Affiliations:** ^1^Department of Cancer Research and Molecular Medicine, Norwegian University of Science and Technology (NTNU), N-7489 Trondheim, Norway, ^2^Computational Biology and Bioinformatics, The Jackson Laboratory, 600 Main Street, Bar Harbor, ME, USA, ^3^Department of Genetics, Stanford University, Stanford, CA, 94305-5120, USA, ^4^UniProt, EMBL-EBI, The Wellcome Trust Genome Campus, Hinxton, Cambridgeshire CB10 1SD, UK, ^5^Department of Technology, Sør-Trøndelag University College, N-7004 Trondheim, Norway and ^6^Department of Biology, Norwegian University of Science and Technology (NTNU), N-7491 Trondheim, Norway

## Abstract

Transcription factors control which information in a genome becomes transcribed to produce RNAs that function in the biological systems of cells and organisms. Reliable and comprehensive information about transcription factors is invaluable for large-scale network-based studies. However, existing transcription factor knowledge bases are still lacking in well-documented functional information.

Here, we provide guidelines for a curation strategy, which constitutes a robust framework for using the controlled vocabularies defined by the Gene Ontology Consortium to annotate specific DNA binding transcription factors (DbTFs) based on experimental evidence reported in literature. Our standardized protocol and workflow for annotating specific DNA binding RNA polymerase II transcription factors is designed to document high-quality and decisive evidence from valid experimental methods. Within a collaborative biocuration effort involving the user community, we are now in the process of exhaustively annotating the full repertoire of human, mouse and rat proteins that qualify as DbTFs in as much as they are experimentally documented in the biomedical literature today. The completion of this task will significantly enrich Gene Ontology-based information resources for the research community.

**Database URL:** www.tfcheckpoint.org

## Introduction

Specific gene regulation mechanisms determine which part of the genome becomes transcribed to provide the active molecular parts of living organisms in various environmental conditions. Central in these mechanisms are multiprotein complexes present at the regulatory regions of genes that determine the onset and rate of RNA synthesis by regulating RNA polymerase activity (1, 2). These multiprotein complexes comprise general transcription factors, general co-factors (3), RNA polymerase II (RNAP II) sequence-specific DNA binding transcription factors (DbTFs) (4) and a large array of transcriptional regulators that lack DNA-binding activity but exert their regulatory roles through protein interaction with the aforementioned proteins (which include co-activators, co-repressors, histone modifiers and chromatin remodeling proteins (1, 2). General transcription factors bind to core-promoter DNA where they constitute pre-initiation transcription complexes, in concert with general co-factors, whereas DbTFs bind to gene-specific proximal and distal gene regulatory regions. RNAP II, one of the three nuclear RNA polymerases (RNAP I, II and III) involved in transcription of mammalian genes, draws special attention in studies directed at gene regulatory mechanisms, as it is responsible for transcribing protein-coding genes as well as miRNA and other RNA genes (5).

Owing to their selective binding within regulatory regions of distinct genes, the DbTFs play decisive roles in directing the assembly of the multiprotein transcription machinery to a particular subset of genes. This assembly can either be followed by immediate RNAP II-dependent transcription or it can result in promoter-proximal pausing of RNAP II that can subsequently be released into active transcription triggered by either DbTFs or other mechanisms (1, 6, 7). DbTFs also play a central role in transcription repression either by competing with activating DbTFs for DNA binding or by recruiting transcriptional co-repressors (2, 8). Through these functions, DbTFs link the phenotypical state of the cell—reflected in abundance and activation state of proteins in the transcriptional machinery—to the decoding of regulatory information embedded within the genome sequence. Thus, the DbTFs are a point of convergence for mechanisms involved in upward causation, i.e. the flow of information from genome to phenome (central dogma), as well as in downward causation, which enables the organism to respond to cues from the extrinsic and intrinsic environment (9).

Current estimates suggest that the human genome contains ∼1900 DbTF-coding genes (10). With the increasing trend to pursue a systems-level understanding of gene regulatory networks (11), it is of key importance to have available genome-wide and accurate information concerning DbTFs including their specific roles in transcription regulation, their target genes (TGs) and their expression patterns related to cell type and to developmental as well as to normal- and pathophysiological processes. This need for genome-wide information has sparked (among others) the ENCODE project, an initiative to identify all functional elements in the human genome sequence and the regulatory interactions between TFs and their transcription factor binding sites (TFBS) (12). Thus, experimental data will continue to become available in ever-increasing volumes, and subsequent comprehensive annotation of functional aspects of DbTFs in public databases will be of high value for ongoing and future gene regulatory studies.

The Gene Ontology (GO) provides a common vocabulary for the functional description of genes and gene products and consists of three sub-ontologies: Biological Process (BP), Molecular Function (MF) and Cellular Component (13). The Gene Ontology Consortium (GOC) provides high-quality classifications for types of transcription factors and captures the supporting evidence for the assignment of classes to gene products. Recently (2010–2011), the GOC undertook a major reorganization of the representation of transcription factors within GO to bring this area up-to-date with current knowledge, to incorporate some advances in the ontological representation allowed in GO and to make all of the transcription factor terms conform to the principle that terms in the MF aspect of GO should represent knowledge about the mechanism of action of that function, e.g. ‘DNA binding’, ‘RNA polymerase binding’ or ‘transcription factor binding’.

The reorganization of the transcription factor MF terms generated a more robust ontology structure by improving both textual definitions and relationships between terms in the ontology structure [(14); see also Supplementary Material 1 for additional comments on background and orientation for the reorganization].

**Figure 1.**
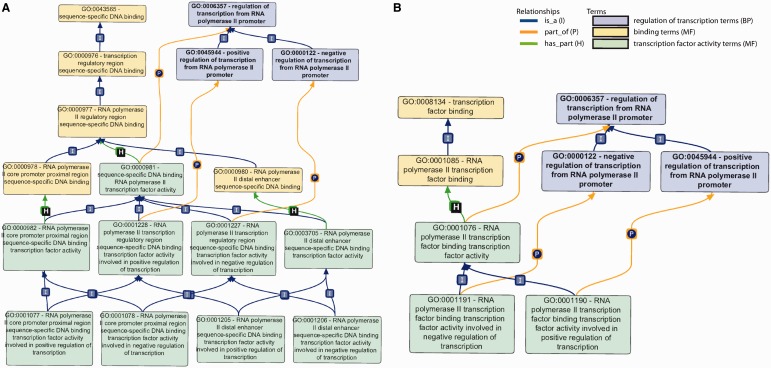
Primary GO terms/subgraphs used for DbTF annotation. (**A**) GO subgraph used for sequence-specific DbTF. In this graph, sequence-specific DNA binding MF terms (yellow), sequence-specific DNA binding TF activity MF terms (green) and transcription regulation BP (blue) are shown along with the relationships between terms in the graph structure. (**B**) GO subgraph used for transcription factor binding transcription factors. In this graph, the different color coding represents the following: TF binding MF terms (yellow), transcription regulation BP (blue) and TF binding TF activity MF terms (green). I, P and H on top of the lines stand for relationships ‘is_a’, ‘part_of’ and ‘has_part’.

For example, nucleic acid-binding transcription factors must have nucleic acid-binding activity to function and also must regulate transcription. Thus, the MF terms for types of ‘nucleic acid binding transcription factor activity’ are required to have ‘has_part’ relationships to the appropriate MF terms for ‘nucleic acid binding’ [e.g. ‘sequence-specific DNA binding RNA polymerase II transcription factor activity’ (GO:0000981) has_part ‘RNA polymerase II regulatory region sequence-specific DNA binding’ (GO:0000977)] (see Figure 1). Equally important, MF ‘transcription factor activity’ terms [e.g. ‘sequence-specific DNA binding RNA polymerase II transcription factor activity’ (GO:0000981)] are also required to have ‘part_of’ relationships to appropriate BP terms for ‘regulation of transcription’ (e.g. ‘regulation of transcription from RNA polymerase II promoter’ (GO:0006357)], as the overall biological objective of the function of the molecule is to take part in regulating transcription. These ‘part_of’ relationships between a specific MF term and a BP term represent a previous advance in the use of relationships within the GO structure to provide more contextually-dependent MF terms, e.g. when the same enzymatic activities are used in more than one process. In the course of revising the transcription section of GO, we incorporated these ‘part_of’ links from MF to BP terms to provide more complete representation of the ‘transcription factor activity’ terms, which are located within the MF aspect of GO. Examples of these ‘has_part’ and ‘part_of’ relationships for these MF terms are shown in Figure 1. Retention of a generic ‘transcription factor activity’ does not make sense in the MF ontology because from a MF viewpoint it is equivalent to an otherwise unknown MF that regulates transcription. However, the BP term ‘transcription, DNA dependent’ can be used to annotate all gene products that regulate transcription, even when the mechanism of action is not known.

Today (GO database release on 16 February 2013), the GOC provides annotations that allow for identification of ∼300 human, mouse and rat DbTFs, which is ∼15% of the expected DbTFs (10). Only ∼200 of these are presently supported by experimental evidence, whereas ∼100 are annotated with evidence based on computational prediction, sequence and structure similarity or author statement (GO database release on 16 February 2013). There are several mammalian DbTF databases, including TFcat (15), JASPAR (16) and TFe (17), that also hold experimentally documented DbTF information based on cited scientific literature. However, these databases lack informative annotations founded on ontologies and evidence codes (as provided by the GOC), which are necessary for rigorous computational reasoning and analysis.

The above findings suggest that, to date, no single comprehensive information resource for mammalian DbTFs exists with the level of coverage and high-quality annotation that is needed for genome-scale data analysis and interpretation. The GOC has standard procedures for annotating proteins, and their database is authoritative in providing comprehensive annotations to the myriad of tools that use GO information for data analysis. However, the capacity of expert curators at the GOC is presently not scaled for or focused for dedicated efforts to comprehensively annotate one particular functional protein class. Therefore, we have embarked on a collaborative effort involving community users and GOC members to exhaustively curate experimentally documented mammalian DbTFs. Similar to other sub-domain annotation initiatives (18, 19), our first aim was to develop specific guidelines for curating experimentally documented DbTFs from literature. This included the assembly of a list of experimental assays that would qualify to provide verifiable functional evidence for genuine DbTFs. Here, we provide a detailed report in the form of a comprehensive curation protocol, based on which we are currently engaged in a focused effort to curate all experimentally characterized DbTFs from a collection of candidate proteins compiled from the major TF information sources. A database providing detailed information about TF information sources and assembled DbTF documentation is available at www.tfcheckpoint.org.

## Creation of annotations for sequence-specific DNA binding RNAPII Transcription Factors (DbTFs)

Our curation guidelines for high-quality annotation of experimentally verified DbTFs are designed to capture the essential functional capabilities of DbTFs and record published evidence using rigorous semantics. In the following sections, we describe fundamental functional characteristics of a DbTF, how these characteristics can adequately be described by GO terms and how these terms and evidence codes can be asserted based on experimental work reported in literature. The assembled procedure facilitates a precise representation of DbTF functional attributes using the standard GOC-defined gene-association file format (GAF2.0; http://www.geneontology.org/GO.format.gaf-2_0.shtml) and the PSI-MI data exchange format used for recording interaction data (20). A detailed DbTF annotation guideline document is provided in Supplementary Material 2.

### Criteria that qualify a DbTF

A DbTF is a DNA binding transcription factor that binds to a specific DNA sequence and regulates the transcription of the associated gene. The specific DNA sequences bound by DbTFs are termed TFBS, and for RNAPII these are located in gene regulatory regions either upstream and proximal to the core promoter, or in more distal upstream or downstream enhancer regions. Once a DbTF recognizes a TFBS, it may recruit other accessory factors or RNAPII, or it may interfere with binding of other regulatory proteins to regulate the expression of the TG. This means that a DbTF must exhibit both DNA-binding and transcription regulation capacity. Therefore, the minimum criteria to qualify a protein as DbTF for RNAPII are that it (i) binds to specific DNA sequences in gene regulatory regions and (ii) is involved in RNAPII-dependent regulation of transcription.

It is evident that to capture these functional aspects accurately and efficiently, the specific GO terms that substantiate these assertions need to be precisely defined. These GO terms must address both ‘sequence-specific DNA binding’ and ‘transcription regulation’ capabilities accurately. In the following sections, we provide a detailed reasoning behind the selection of specific GO terms of different granularity as well as assignment of GO evidence codes and experimental assays that are considered adequate and necessary for creating a DbTF annotation.

### GO terms used for DbTF annotation

#### Specific DNA binding

To capture the capability of a protein to bind to specific DNA sequences, a GO MF term that describes ‘sequence-specific DNA binding’ (e.g. GO:0043565 ‘sequence-specific DNA binding’) should be used. GO:0000976 ‘transcription regulatory region sequence-specific DNA binding’ should be used when it is not possible to identify information stating that the regulatory region containing the DNA sequence specifically bound by the DbTF is part of a gene regulated by RNAP II. Where a gene is known to be transcribed by RNAP II, a more specific term (GO:0000977 ‘RNA polymerase II regulatory region sequence-specific DNA binding’) may be applied. If information exists that indicates whether the protein binds the proximal or distal regulatory regions, this may be indicated by use of either of the terms describing the location of binding (GO:0000978 ‘RNA polymerase II proximal region sequence-specific DNA binding’ or GO:0000980 ‘RNA polymerase II distal enhancer sequence-specific DNA binding’) (Figure 1A, terms shaded yellow).

#### Transcription regulation

The involvement of a protein in transcription regulation is well captured by the GO BP terms GO:0006357 (regulation of transcription from RNAP II promoter) or any of its children that specify whether the protein is involved in positive or negative regulation of transcription (Figure 1A, terms shaded blue).

#### Sequence-specific DNA binding RNAP II transcription factor activity

The goal of this curation project is to assign a sequence-specific DbTF activity term, i.e. GO:0000981 (sequence-specific DNA binding RNAP II transcription factor activity) or one of its children to appropriate DbTFs (Figure 1A, terms shaded green). As indicated above, this requires that the composite functional aspects of DbTF proteins—specific DNA binding and transcription regulation— must each be represented by their proper MF and BP GO terms. These different aspects of DbTF activity— specific DNA binding and involvement in transcriptional regulation—are typically demonstrated in different experiments, sometimes not even presented in the same paper, so the annotations to specific DNA binding (MF) and transcriptional regulation (BP) terms are made separately, and only when both are assigned (each in their inherent logic of the GO-structure) can they be combined to infer DbTF activity MF terms (Table 1).

**Table 1.** Inference of transcription factor activity terms from DNA/TF binding and transcription regulation terms

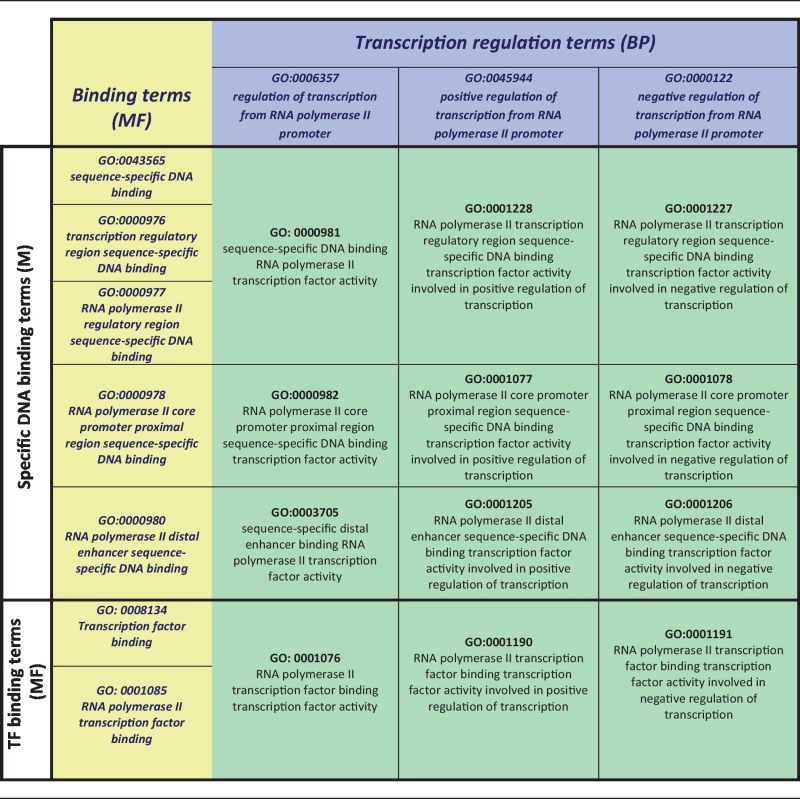

Each transcription factor activity term (green) is determined by the composite annotation of the corresponding DNA binding or TF binding term (yellow) and a transcription regulation term (blue).

The child terms of ‘GO: 0000981’ are used to delineate whether the TF exerts its activity by binding to the promoter proximal region or the distal enhancer, i.e. ‘GO:0000982 (RNAP II core promoter proximal region sequence-specific DbTF activity)’ or ‘GO:0003705 (sequence-specific distal enhancer binding RNAP II transcription factor activity)’, and whether the result of binding is positive or negative regulation of TG transcription, e.g. ‘GO:0001077 (RNAP II core promoter proximal region sequence-specific DbTF activity involved in positive regulation of transcription)’ and ‘GO:0001205 (RNAP II distal enhancer sequence-specific DbTF activity involved in positive regulation of transcription)’. Genes that have been shown to have both positive and negative regulatory roles should be annotated with both the positive and negative regulation terms as appropriate.

#### TF binding and TF binding TF activity

Transcriptional regulation mechanisms are complex. Usually many TFs work together in concert to regulate transcription. In instances where the activity of a TF is reported to be dependent on interaction with another protein or multi-subunit complex, the protein–protein interaction (PPI) is annotated using ‘transcription factor binding’ MF GO terms as shown in Figure 1B (terms shaded yellow). Furthermore, a different set of ‘transcription factor activity’ terms, i.e. ‘GO:0001076 (RNAP II transcription factor binding transcription factor activity)’ or any of its children, is chosen to reflect the fact that the activity is dependent on binding to another TF (Figure 1B, terms shaded green).

Once TF binding and transcription regulation are each annotated individually, the GO structure allows for the generation of TF binding TF activity annotations by combining the separate annotations (Table 1).

### When the functional unit of a TF is a complex

In instances where the complex is a homodimer or higher order multimer of the same protein, there are no special annotation issues, as all of the activities demonstrated are properties of the same gene product. However, when the functional unit is a heterodimer or other multisubunit complex, then there are some additional considerations for annotation.

The ‘contributes_to’ qualifier is specifically intended for the annotation of functions that occur in the context of complexes, rather than being an activity of a single subunit within the complex. In the case of a heterodimer, there are times where one of the two proteins does not bind DNA on its own. However, in some cases, a subunit that does not bind DNA independently can be shown to contribute to the sequence specificity of binding when present within a heterodimer. In this situation, the subunit that does not bind DNA alone could be annotated to appropriate ‘sequence-specific DNA binding’ terms (Figure 1A, terms shaded yellow) using the qualifier ‘contributes_to’ to indicate that it contributes to the DNA binding of the heterodimer. More generally, the ‘contributes_to’ qualifier can be used in conjunction with any MF term, including the ‘transcription factor activity’ terms, to indicate that it contributes to that function within the context of a complex, even though it does not possess that activity independently. In contrast, in a multisubunit TF where the DNA binding activity is known to be confined to one or more specific subunits, other subunits should not be annotated to a ‘specific DNA binding’ term at all.

For any subunit within a TF complex, it is appropriate to annotate all appropriate GO terms for which that function has been experimentally shown, either individually or as part of the complex indicated with the ‘contributes_to’ qualifier. Thus, in some cases, a given protein may be annotated both with a ‘sequence-specific DNA binding RNAP II transcription factor activity’ term as well as with a ‘TF binding RNAP II transcription factor activity’ term.

### Evidence codes and experimental assays

In accordance with the overall guidelines for GO annotations, each DbTF annotation must be qualified with an evidence code indicating how the annotation is supported by experimental evidence (http://www.geneontology.org/GO.evidence.shtml). The DbTF curation guidelines presented in the current work use one of the following GO evidence codes: Inferred from Direct Assay (IDA), Inferred from Physical Interaction (IPI), Inferred from Mutant Phenotype (IMP) or Inferred by Curator (IC).

When a single scientific paper comprises all experimental evidence necessary to support each of the annotations for ‘DNA- or TF-binding’ and ‘Transcription regulation’, the evidence codes for these two annotations are transferred to the composite DbTF annotation to a MF ‘transcription factor activity’ term (see Table 2). However, when the two different types of annotations (‘DNA’ or ‘TF-binding’ and ‘transcription regulation’) for a given TF cannot be generated from one single paper, the evidence code IC is used along with the GOC-generated reference, GO_REF:0000036 (http://www.geneontology.org/cgi-bin/references.cgi#GO_REF:0000036). The IC code, which requires the use of the two GO IDs for the appropriate ‘binding’ and ‘transcription regulation’ terms, indicates that GO annotations based on evidence from two different sources have been combined by a curator to infer the appropriate transcription factor activity term.

**Table 2.** Evidence code table

DNA binding/ TF binding	Transcription regulation	TF activity
IDA	IDA	IDA/IC^a^
IMP	IMP	IMP/IC^a^
IDA	IMP	IDA, IMP/IC^a^
IMP	IDA	IMP, IDA/IC^a^
IPI^b^	IDA	IPI^b^, IDA/ IC^a^

IC^a^ if evidence for ‘DNA binding / TF binding’ and ‘transcription regulation’ comes from two different papers.

IPI^b^ applicable only for TF binding terms.

To provide for a uniform standard for evaluation of experimental evidence for DbTF annotations, we surveyed several relevant resources defining experimental assays that can document TF function, including ORegAnno (21), TRRD (22), RegulonDB (23) and the PSI-MI controlled vocabulary for molecular interactions (20).

In the following sections, we have compiled sets of selected experimental assays that we deem to be most relevant for annotation of DNA binding, TF binding and transcription regulation. PSI-MI-unique identifiers are given wherever they exist. Augmentation of the PSI-MI vocabulary to span a larger repertoire of TF-defining experiments is ongoing.

#### Specific DNA binding

Experimental data documenting specific DNA binding are obtained from experiments that show *in vitro* binding of a TF to specific DNA sequences present in either cloned TG regulatory regions (proximal promoter and/or distal enhancer) or in synthetic DNA sequences representing canonical TF binding sites or specific TG regulatory regions (see Table 3). We have chosen not to rely on assays measuring *in vivo* TF–DNA interaction (e.g. the Chromatin ImmunoPrecipitation assay) because it is not possible to ascertain in these assays that the TF in question actually binds directly to DNA, or whether some other component in the *in vivo* system mediates the TF–DNA association.

**Table 3.** Assays documenting specific DNA binding

Experimental assays	Variants	Evidence code	PSI-MI code
EMSA	Nuclear extract from native tissue or cells	No evidence	MI:0413
	Nuclear extracts from cells or tissue with ectopic expression of a TF	IDA	MI:0413
	Purified TF (*in vitro* translated or purified from cell extract)	IDA	MI:0413
	Nuclear extract from cells with ectopic expression of a mutated TF	IMP	MI:0413
	Purified mutated TF (*in vitro* translated or purified from cell extract	IMP	MI:0413
Electrophoretic mobility supershift assay (EMSA supershift)	Nuclear extract from native tissue or cells	IDA	MI:0412
	Nuclear extracts from cells or tissue with ectopic expression of a TF	IDA	MI:0412
	Purified TF (*in vitro* translated or purified from cell extract)	IDA	MI:0412
	Nuclear extract from cells with ectopic expression of a mutated TF	IMP	MI:0412
	Purified mutated TF (*in vitro* translated or purified from cell extract)	IMP	MI:0412
Footprinting		IDA	MI:0417
DNase I footprinting (DNA footprint)		IDA	MI:0606
Methylation interference assay (MIC)		IDA	MI:1189
Ultraviolet (UV) footprinting (UV-footprint)		IDA	MI:1191
Dimethylsulphate footprinting (DMS-footprint)		IDA	MI:0603
Hydroxy radical footprinting (Hydroxy-footprint)		IDA	MI:1190
Potassium permanganate footprinting (KMnO4-footprint)		IDA	MI:0604
Affinity chromatography technology		IDA	MI:0004
Pull down		IDA	MI:0096
Southwestern blot assay (SW-blot)		IDA	
*In vitro* evolution of nucleic acids (SELEX)		IDA	MI:0657
X-ray crystallography		IDA	MI:0114

The experimental assays are denoted with their standard nomenclature in PSI-MI; for the detailed description please see: http://www.ebi.ac.uk/ontologylookup/browse.do?ontName=MI. For Southwestern blot assay, see: http://www.nlm.nih.gov/mesh/.

The *in vitro* assay that has been most frequently used for documenting sequence-specific binding of TF is the Electrophoretic Mobility Shift Assay (EMSA) (24). The most common variants of this assay present the TF in the form of:

- nuclear extract from native tissue or cells
- nuclear extracts from cells or tissue with ectopic expression of a TF
- purified TF (*in vitro* translated or purified from cell extract)
- nuclear extract from cells with ectopic expression of a mutated TF
- purified mutated TF (*in vitro* translated or purified from cell extract).

When the TF is presented in any of the variants (ii–v), the EMSA qualifies for annotation of a GO term for ‘specific DNA binding’. In the case where the TF is presented as a nuclear extract from native cells or tissue (i), we require that the specific TF is identified with an additional experimental approach. This may involve specific competition experiments demonstrating that the EMSA gel shift is not abolished by competition with an unlabeled DNA probe with a point mutation in a known TFBS for this specific TF, whereas competition with unlabeled DNA probe containing the wild-type TFBS does abolish the gel shift. Also, the use of a TF-specific antibody, i.e. EMSA supershift, will increase confidence in EMSA assays with nuclear extracts from native tissue or cells; however, these assays must be interpreted with caution, as the DNA–protein complex may be shifted even though a different protein than the one recognized by the antibody provides for the DNA-binding part in the complex. If no additional experimental verification of the TF is reported, nuclear extract-based EMSAs of type (i) do not suffice to qualify DNA binding properties of a TF, and the experiment needs to be dismissed.

Similarly, the other assays listed in Table 3 must have been performed in a manner that provides for identification of the specific TF tested and to assess specific interaction between this TF and a specified DNA probe. For MI:0114 X-ray crystallography, to qualify as experimental evidence of a TFs DNA binding, it is required that the protein is co-crystallized with a DNA sequence that represents either a canonical TFBS or an authentic gene regulatory region.

#### Transcription regulation

The ‘transcription regulation’ terms need support from assays that document modulation of transcriptional process in response to TF action*.* These assays mainly fall into two groups: either reporter gene assays measuring the transcriptional regulatory effect of a TF on a regulatory region cloned upstream of a reporter gene (e.g. luciferase, beta-galactosidase or chloramphenicol acetyltransferase), or measurement of expression levels of a TG mRNA (see Table 4). Within each of the assays, a variety of experimental strategies can allow for the identification of the specific TF [e.g. ‘knock in’ (ectopic expression) and/or ‘knock down’]. Furthermore, the gene regulatory region can be presented and assessed in different ways in the reporter gene assays (e.g. ‘canonical TFBS’ or ‘authentic TG promoter/enhancer’) and different methods used to assay mRNA expression levels of specific TGs. The combinations of different modes of TF and TG detection together define the GO evidence codes to be used (Table 4).

**Table 4.** Reporter gene-based assays variants documenting transcription regulation

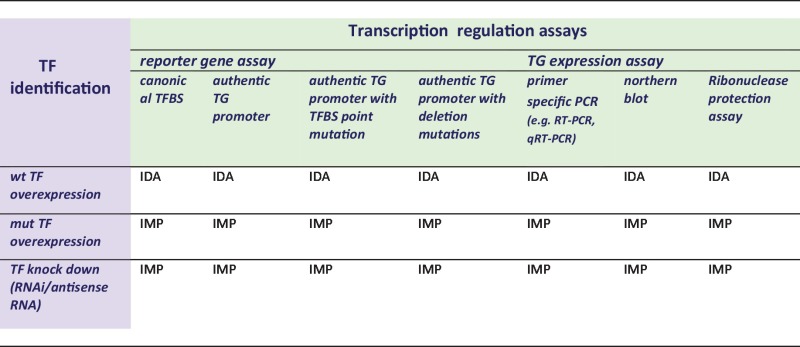

This table is a decision matrix for selecting GO evidence codes based on the method used for TF identification (purple) and transcription regulation (green). wt = wild type, mut = mutated.

Although the experimental assays depicted in Table 4 are most often carried out by transfecting expression and reporter plasmids into cell line model systems, transcription regulation annotations can also be supported by whole organism experiments, e.g. knock out mutations or RNAi knock down strategies. However, as such experiments do not by themselves prove a role in regulation of transcription, such annotations must be made with caution and will depend on a strict awareness of additional information such as the concomitant documentation of specific binding by the protein in question, to regulatory regions of an RNAP II regulated gene (e.g. by Chromatin ImmunoPrecipitation assay analysis).

#### TF binding

‘TF binding’ specific terms are based on any assay that provides evidence for PPIs. Table 5 lists experimental assays and evidence codes that are eligible for TF binding specific terms. Currently, we are only making these annotations from ‘small scale’ papers that we come across for proteins that are also DbTFs or for proteins with sequence similarity to DbTFs but which do not appear to bind DNA. Any future extension of this work to use high throughput PPI data would need to carefully consider what standards should be applied to minimize the effect of the high level of false-positives in high throughput PPI data.

**Table 5.** Assays documenting TF binding

Assays	Evidence code	PSI-MI code
2-Hybrid interactions	IPI	MI:0018
Co-purification	IPI, IDA	MI:0004
Co-immunoprecipitation	IPI, IDA	MI:0019

The experimental assays are denoted with their standard nomenclature in PSI-MI; for the detailed description please see: http://www.ebi.ac.uk/ontologylookup/browse.do?ontName=MI.

## Annotating TGs

An obvious important biological property of a TF lies in the particular TGs that it regulates. Proper recording of this information is of key importance for the building of gene regulatory networks. In studies of DbTF functionality, often one or several specific TGs will be identified and experimentally documented. The GOC has introduced an Annotation Extension field to capture additional information that provides more biological context to the GO annotation (GAF 2.0, http://www.geneontology.org/GO.format.gaf-2_0.shtml). This field can be used to record information regarding specific TGs regulated by the TF that is being annotated. The TG is recorded in the Annotation Extension field for the BP transcription regulation GO term using the ‘has_regulation_target’ relationship combined with the gene identifier(s) for the TG(s).

## Work flow of annotation

**Figure 2.**
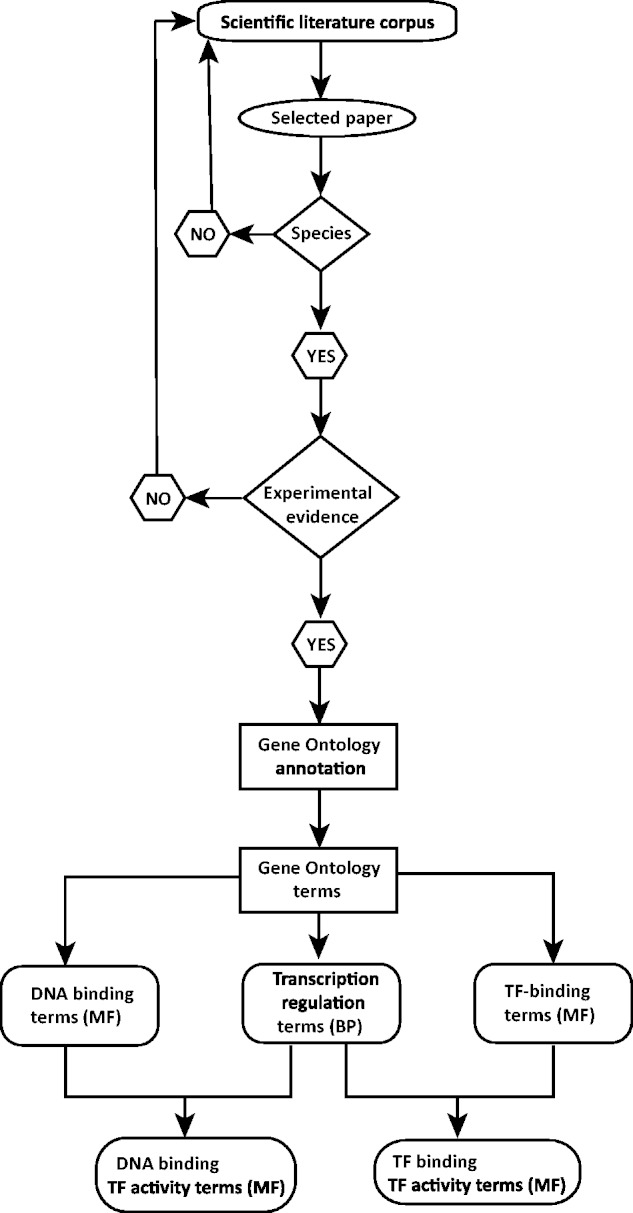
Sequence-specific DNA binding TF (DbTF) curation workflow. This workflow represents the step-by-step procedure for curating experimentally verified mammalian DbTFs from scientific publications. Selection of scientific publication from the literature corpus is the starting point of the curation procedure. From each relevant publication, DbTF-specific GO-terms are annotated and recorded.

**Figure 3.**
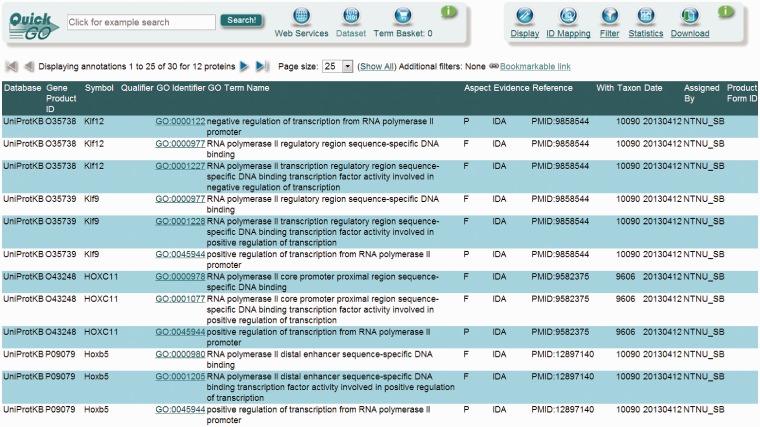
UniProt-GOA screenshot of some of the DbTF annotations. The annotations generated using the DbTF curation guidelines discussed here can be accessed from the GO database using the QuickGO tool.

The annotation workflow is depicted in Figure 2. An annotation effort typically starts with one of the scientific papers suggested in databases such as TFCat and JASPAR to document a candidate DbTF, or by searching for adequate literature in one of the following resources: UniProt (http://www.uniprot.org/), NCBI’s Entrez Gene (25), iHOP (26), Gene Cards (27) or NCBI’s PubMed (http://www.ncbi.nlm.nih.gov/pubmed/). Each scientific paper is first checked for information providing correct identification of species origin of the TF studied. Because we are focusing on DbTFs from human, mouse and rat studies, only papers allowing identification of a DbTF from one of these species will proceed to further curation. Thus, a number of papers that fail to clearly identify the species of the gene(s) used in their construct(s) have to be omitted from the curation process. Then, the paper is searched for adequate experimental evidence to support one or several DbTF annotations. If either TF species origin or sufficient experimental evidence is not identifiable, the curator returns to the scientific literature corpus to search for other suitable papers. When both criteria are fulfilled, the individual GO annotations (i.e. specific DNA binding and/or TF binding and transcription regulation) are assigned together with a supporting evidence code. Finally, the composite TF activity MF GO term(s) is inferred. TF annotation data are submitted to UniProt-GOA in the form of a gene association file (GAF2.0; http://www.geneontology.org/GO.format.gaf-2_0.shtml) and will subsequently appear in the GOC database via tools such as AmiGO (http://amigo.geneontology.org/) and QuickGO (http://www.ebi.ac.uk/QuickGO/; Figure 3).

## DISCUSSION

### Benefits of a focused annotation project

A comprehensive resource of high-quality annotations of TFs is of high value both for small-scale experiments where it is important to select an optimal subset of relevant TFs and for genome-scale studies. In the latter case, access to extensive background knowledge for TFs is essential to infer gene regulatory networks (28) or to design experiments to characterize this group of proteins as a functional class in a system-wide approach (29, 30).

Compilation and in-depth analysis of available information on transcription factors indicate that >800 mammalian DbTFs are experimentally documented in the scientific literature (www.tfcheckpoint.org). The current work aims to provide the foundation to curate this source of information and to record adequate GO annotations in compliance with the standards defined here. Currently (GO database release on 16 February 2013), only 202 human, mouse and rat proteins are annotated as DbTFs with ‘GO:0000981 sequence-specific DNA binding RNA polymerase II transcription factor activity’ (or any of its child terms) supported by experimental evidence, meaning that some 600 DbTFs still need to be processed. We aim to complete this task before the end of 2013. Even though the number of curators involved is small, the efficiency of this focused annotation project is high, as the number of different GO terms and evidence codes is limited and well defined, thus allowing each curator to process a relatively high number of scientific papers (typically five papers or more per working day).

### Added value of rigorous classification of experimental assay requirements for the annotations

The catalogue of experimental assays that qualify for supporting TF annotations presented here is assembled based on the extensive TF annotation experience in the collaborating organizations. This aspect of the annotation procedure improves the quality of the GO annotations, as it provides a uniform standard for interpretation of evidence strength in published experimental work. As some of the assays presently are not adequately covered by PSI-MI vocabulary (20), a part of our efforts has been directed to collaborate with the PSI-MI consortium to develop additional PSI-MI terms. The proper documentation of experimental evidence for each TF annotation will enable us to work toward submitting annotated data to the IntAct database (31). Moreover, we plan to make the experimental assay details for the TF annotations available to users via our TF database (http://www.tfcheckpoint.org/). This will enable users to select subsets of TFs based on the specific experimental methods used to characterize them.

## Concluding remarks

Metadata are rarely presented in biomedical publications using formalized knowledge representation. This often makes it difficult for a curator to extract accurate information for ontology- or structured vocabulary-annotation from natural language used in the literature. The GOC provides guidelines for the curation of gene products information from scientific publications and procedures for identification of the type of evidence that supports the curated information. Because of these standardized conventions, literature-curated data in the GO database are deemed to be of high utility and quality. In the present work, we have established a comprehensive and specific curation procedure for TFs of RNAP II, which, similar to other data standardization initiatives, provides details on the requirements to properly record an experimentally verified DbTF.

The GOC is centrally involved in efforts to provide annotation guidelines for particular protein functional categories. However, the elaboration of procedures for specific tasks like the curation of distinct functional categories of proteins, or of BP subdomains, is enhanced when experts in the respective fields are involved in the curation process. Moreover, the active participation from domain experts is greatly facilitated by generating detailed curation guidelines as vehicles for productive interactions. With the transcription factor curation effort presented here, we wish to provide not only a greater number of high-quality annotations for DbTFs and their TGs across three mammalian species, but also to exemplify the constructive use of detailed guidelines to facilitate collaborative biocuration efforts across institutions.

## Funding

The Norwegian Cancer Society, The Liaison Committee between the Central Norway Regional Health Authority (RHA), the Norwegian University of Science and Technology (NTNU) and Sør- Trøndelag University College (HisT). K.R.C., J.A.B., D.P.H. and R.B. are funded by NHGRI grant (HG 002273) for the Gene Ontology Consortium. R.H. is funded by NHGRI grant NIH (U41HG002273). Funding for open access charge: Norwegian University of Science and Technology.

*Conflict of interest*. None declared.

## Supplementary Material

Supplementary Data

## References

[1] Weake VM, Workman JL (2010). Inducible gene expression: diverse regulatory mechanisms. Nat. Rev. Genet..

[2] Perissi V, Jepsen K, Glass CK, Rosenfeld MG (2010). Deconstructing repression: evolving models of co-repressor action. Nat. Rev. Genet..

[3] Thomas MC, Chiang CM (2006). The general transcription machinery and general cofactors. Crit. Rev. Biochem. Mmol. Biol..

[4] Mitchell PJ, Tjian R (1989). Transcriptional regulation in mammalian cells by sequence-specific DNA binding proteins. Science.

[5] Lee Y, Kim M, Han J (2004). MicroRNA genes are transcribed by RNA polymerase II. EMBO J..

[6] Rahl PB, Lin CY, Seila AC (2010). c-Myc regulates transcriptional pause release. Cell.

[7] Adelman K, Lis JT (2012). Promoter-proximal pausing of RNA polymerase II: emerging roles in metazoans. Nat. Rev. Genet..

[8] Thiel G, Lietz M, Hohl M (2004). How mammalian transcriptional repressors work. Eur. J. Biochem..

[9] Noble D (2012). A theory of biological relativity: no privileged level of causation. Interface Focus.

[10] Vaquerizas JM, Kummerfeld SK, Teichmann S, Luscombe NM (2009). A census of human transcription factors: function, expression and evolution. Nat. Rev. Genet..

[11] Walhout AJM (2006). Unraveling transcription regulatory networks by protein-DNA and protein-protein interaction mapping. Genome Res..

[12] Neph S, Vierstra J, Stergachis AB (2012). An expansive human regulatory lexicon encoded in transcription factor footprints. Nature.

[13] Gene Ontology Consortium (2013). Gene Ontology annotations and resources. Nucleic Acids Res..

[14] Leonelli S, Diehl AD, Christie KR (2011). How the gene ontology evolves. BMC Bioinformatics.

[15] Fulton DL, Sundararajan S, Badis G (2009). TFCat: the curated catalog of mouse and human transcription factors. Genome Biol..

[16] Sandelin A, Alkema W, Engström P (2004). JASPAR: an open-access database for eukaryotic transcription factor binding profiles. Nucleic Acids Res..

[17] Yusuf D, Butland SL, Swanson MI (2012). The transcription factor encyclopedia. Genome Biol..

[18] Inglis DO, Skrzypek MS, Arnaud MB (2013). Improved gene ontology annotation for biofilm formation, filamentous growth, and phenotypic switching in Candida albicans. Eukaryotic cell.

[19] Mutowo-Meullenet P, Huntley RP, Dimmer EC (2013). Use of Gene Ontology Annotation to understand the peroxisome proteome in humans. Database.

[20] Hermjakob H, Montecchi-Palazzi L, Bader G (2004). The HUPO PSI’s molecular interaction format—a community standard for the representation of protein interaction data. Nat. Biotechnol..

[21] Griffith OL, Montgomery SB, Bernier B (2008). ORegAnno: an open-access community-driven resource for regulatory annotation. Nucleic Acids Res..

[22] Kolchanov N, Ignatieva EV, Ananko E (2002). Transcription Regulatory Regions Database (TRRD): its status in 2002. Nucleic Acids Res..

[23] Gama-Castro S, Jiménez-Jacinto V, Peralta-Gil M (2008). RegulonDB (version 6.0): gene regulation model of Escherichia coli K-12 beyond transcription, active (experimental) annotated promoters and Textpresso navigation. Nucleic Acids Res..

[24] Woo AJ, Dods JS, Susanto E (2002). A proteomics approach for the identification of DNA binding activities observed in the electrophoretic mobility shift assay. Mol. Cell. Proteomics.

[25] Maglott D, Ostell J, Pruitt KD, Tatusova T (2011). Entrez Gene: gene-centered information at NCBI. Nucleic Acids Res..

[26] Hoffmann R, Valencia A (2004). A gene network for navigating the literature. Nat. Genet..

[27] Safran M, Solomon I, Shmueli O (2002). GeneCards 2002: towards a complete, object-oriented, human gene compendium. Bioinformatics.

[28] Ye C, Galbraith SJ, Liao JC, Eskin E (2009). Using network component analysis to dissect regulatory networks mediated by transcription factors in yeast. PLoS Comput. Biol..

[29] Choi MY, Romer AI, Hu M (2006). A dynamic expression survey identifies transcription factors relevant in mouse digestive tract development. Development.

[30] Gray P, Fu H, Luo P (2004). Mouse brain organization revealed through direct genome-scale TF expression analysis. Science.

[31] Kerrien S, Aranda B, Breuza L (2012). The IntAct molecular interaction database in 2012. Nucleic Acids Res..

