# Supplementary material for: Gene Ontology annotation of sequence-specific DNA binding transcription factors: setting the stage for a large-scale curation effort
Source: Database (Oxford). 2013 Aug 27;2013:bat062. doi: 10.1093/database/bat062 (PMC3753819; doi:10.1093/database/bat062)
Supplement: Supplementary Data [file supp_2013_bat062_index.html]

Supplementary Data 

# Gene Ontology annotation of sequence-specific DNA binding transcription factors: setting the stage for a large-scale curation effort

## Supplementary Data

files

**Files in this Data Supplement:**

- Supplementary Data - docx file
- Supplementary Data - docx file
