# Supplementary material for: Gene Ontology annotation of sequence-specific DNA binding transcription factors: setting the stage for a large-scale curation effort
Source: Database (Oxford). 2013 Aug 27;2013:bat062. doi: 10.1093/database/bat062 (PMC3753819; doi:10.1093/database/bat062)
Supplement: Supplementary Data [file supp_bat062_Supplementary_material_1.docx]

**Supplementary material 1**

**Reorganization of transcription factor Gene Ontology structure – additional comments on background and orientation**

After a thorough review of the literature and consultation with experts, it became clear that the term “transcription factor” could not be restricted to a singular molecular function representing DNA-binding transcription factors. Molecular functions are used to describe the molecular mechanisms by which gene products act. While sequence specific DNA binding transcription factors may represent the function of a majority of gene products described as ‘transcription factors’, there are other proteins called ‘transcription factors’ that do not bind DNA. These “protein-binding transcription factors” include some of the general transcription factors (GTFs) for RNA polymerase II and also proteins that bind to DNA binding transcription factors to positively or negatively regulate that DNA binding activity and thus the overall transcription factor activity; some of this latter class are in gene families with DNA binding transcription factors but have lost the DNA binding activity (1) or are generated by alternative splicing of genes that also produce proteins with DNA binding activity (2, 3). This conflation of two mechanisms of action into a single nomenclature required the top level MF terms representing transcription factors to be disambiguated regarding whether the factor regulates transcription via nucleic acid-binding or via protein-binding action. In contrast, Biological Process terms represent a series of molecular functions that contribute to an overall biological objective. Thus the BP term “transcription, DNA-dependent” can be used to identify all gene products with an annotated role in transcription even if the mechanism of their action is not known.

Similarly, specifically within the area of the DNA binding transcription factors, our review of the literature and discussion with experts revealed a great deal of ambiguity and variability in the usage of terminology used to refer to various portions of the transcription regulatory region, especially across the full scope of the transcription literature including that of prokaryotes (e.g. *E. coli*), Archaea, organellar RNA polymerases (mitochondrial and chloroplast), the vast quantity of RNA polymerase II literature spanning a broad range of eukaryotes from *S. cerevisiae* to mammalian species, and also the other nuclear RNA polymerases (I, III, and also IV and V in plants). Most significantly, the word “promoter” is used for two mutually exclusive meanings. The first, and probably original meaning, as commonly used in the *E. coli* literature but also used in some RNA polymerase II literature, is a strict usage referring only to the exact site where the RNA polymerase “holoenzyme” binds. The second usage, which is common in the RNA polymerase II literature, is much broader and generally encompasses the entire proximal regulatory region, both the “core promoter” (which corresponds to the meaning of the strict usage of “promoter”) and the entire regulatory region that is proximal to and usually upstream of the “core promoter”, some portions of which may be referred to as proximal enhancers, depending on the species and specific RNA polymerase. In the absence of a universal and unambiguous terminology across the span of RNA polymerase research, we chose to use the phrase “transcription regulatory region” to cover the entire span of the nucleic acid region(s) regulating transcription. This region may include the “core promoter”, the “core promoter proximal region”, and “distal enhancers”, as appropriate for the species and polymerase. Where possible, we have added phrases more commonly used in the literature as synonyms for our unambiguous ontological names, though additional phrases can be added as is useful.

1. Kee,B.L. (2009) E and ID proteins branch out. *Nature reviews. Immunology*, **9**, 175–84.

2. Liu,Y., Bernard,H.U. and Apt,D. (1997) NFI-B3, a novel transcriptional repressor of the nuclear factor I family, is generated by alternative RNA processing. *The Journal of biological chemistry*, **272**, 10739–45.

3. Sadamoto,H., Saito,K., Muto,H., Kinjo,M. and Ito,E. (2011) Direct observation of dimerization between different CREB1 isoforms in a living cell. *PloS one*, **6**, e20285.
