# Supplementary material for: Gene Ontology annotation of sequence-specific DNA binding transcription factors: setting the stage for a large-scale curation effort
Source: Database (Oxford). 2013 Aug 27;2013:bat062. doi: 10.1093/database/bat062 (PMC3753819; doi:10.1093/database/bat062)
Supplement: Supplementary Data [file supp_bat062_Supplementary_material_2_R2.docx]

**Supplementary material 2**

**Curation Guidelines**

- **Sequence-specific DNA binding RNA polymerase II transcription factors**
- **Transcription factor binding RNA polymerase II transcription factors**

| ***Content*** | ***Page*** | |
| --- | --- | --- |
| **Curation work flow** | 1 |  |
| **Create TF annotations** |  |  |
| Sequence-specific DNA binding (MF) | 2 |  |
| Transcription regulation (BP) | 3 |  |
| Sequence-specific DNA binding TF activity (MF) | 4 |  |
| Evidence codes for DNA binding TF activity | 5 |  |
| TF binding TF activity | 6 |  |
| When the functional unit of a TF is a complex | 7 |  |
| **Create TG annotations** | 8 |  |
| **GO terms with definitions** |  |  |
| Transcription regulation(BP) | 9 |  |
| Sequence-specific DNA binding (MF) | 9 |  |
| Sequence-specific DNA binding TF activity (MF) | 10 |  |
| TF binding activity and process (MF, BP) | 11 |  |
| **GO Evidence Codes with definitions** | 12 |  |
|  |  |  |
| **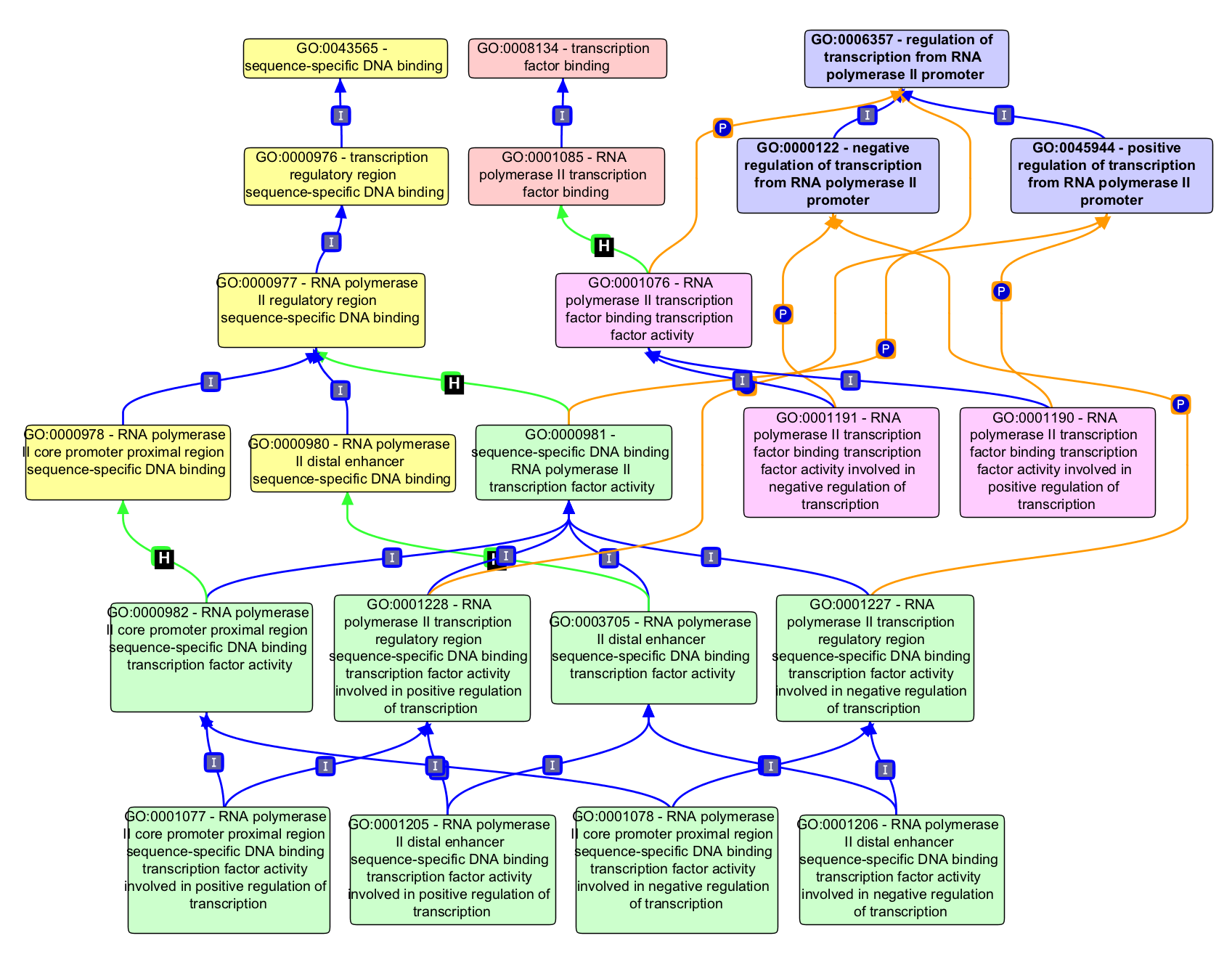** | **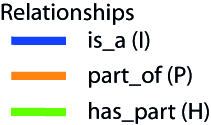** |  |

**Figure 1: Primary GO terms/subgraphs used for DbTF annotation.** In this graph, sequence-specific DNA binding MF terms (yellow), sequence-specific DNA binding TF activity MF terms (green), TF binding MF terms (orange), transcription regulation BP (blue) and TF binding TF activity MF terms (pink) are shown along with the relationships between terms in the graph structure.

**Curation workflow**

| **Identify TF species**  The current curation guidelines are focused on the mammalian species: human, mouse, rat  The species that the TF (i.e. its coding sequence) originates from must be unequivocally determined.  If the publication used for curation does not state this explicitly, TF references must be traced for species determination or authors contacted to obtain relevant information.  **If it is not possible to assert the species of the TF studied, then the paper cannot be used for curation.** | 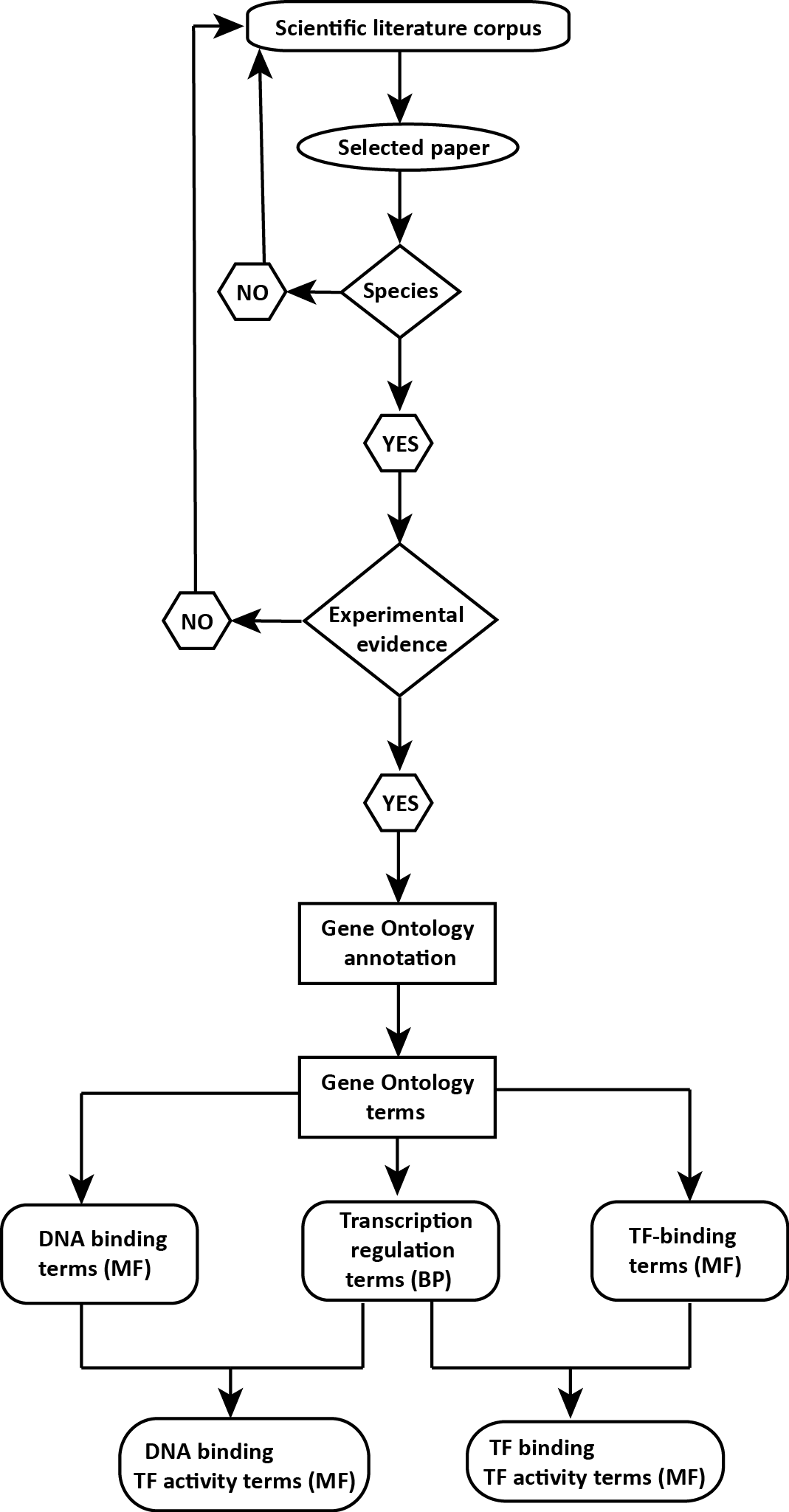  **Figure 2: Sequence - specific DNA binding**  **TF (DbTF) curation workflow** |
| --- | --- |
| **Create annotations for DNA binding, Transcription Regulation, TF binding**  Identify an experiment that qualifies for annotation.  Use Assay look-up Tables (pages 2 - 6) to assess eligibility and GO evidence code.  (optional) **Assign experimental assay term and PSI-MI code**  Use Assay look-up Tables (pages 2 - 6) to assign experimental assay, its variant and PSI-MI code |  |
| **Create annotations for DbTF activity or TF binding activity**  Create these terms by combining the DNA (or TF) binding and transcription regulation terms generated in the steps above.  Use ‘*Decision Tables’* for DNA binding TFs (Table 3, page 4) or TF binding TFs (Table 6, page 6) |  |
|  |  |
|  |  |

**Sequence-specific DNA binding annotations**

| Use Table 1 to identify valid experimental evidence for one of the GO terms for DNA binding shown in Figure 3  Whenever possible, choose *GO:0000977 - RNA polymerase II regulatory region sequence-specific DNA binding* or one of its children. Use *GO:0043565* only when it is not possible to identify information stating that the specific DNA sequence bound by the protein is found in a gene regulatory region, and *GO:0000976* only when it is not possible to identify information stating that the regulatory region containing the DNA-sequence specifically bound by the protein is part of a gene regulated by RNA polymerase II.  DNA binding detection methods differ in how the TF is presented (as detailed for EMSA variants in Table 1). Presentation of TF as a nuclear extract from native cells or tissue is not sufficient for annotation of DNA binding *(“No evid.”).* In these instances we search for other experimental evidence that can identify the specific TF, e.g. TF-specific antibody in EMSA supershift.  X-ray crystallography used as evidence for DNA binding requires that the TF is co-crystallized with a DNA sequence that represents either a canonical TFBS or an authentic gene regulatory region. | 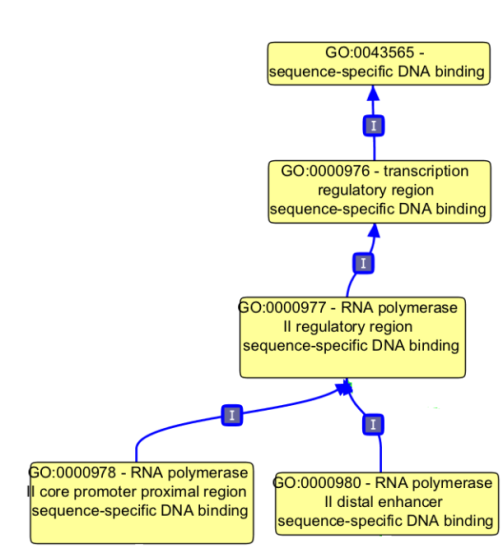  **Figure 3: GO terms for MF specific DNA binding** |
| --- | --- |

| **Table 1: Assays documenting specific DNA binding.**   \| Experimental assays \| Variants \| Evidence code \| PSI-MI code \| \| --- \| --- \| --- \| --- \| \| Electrophoretic mobility shift assay (EMSA) \| nuclear extract from native tissue or cells \| *No evid.* \| MI:0413 \| \|  \| nuclear extracts from cells or tissue with ectopic expression of a TF \| IDA \| MI:0413 \| \|  \| purified TF (*in vitro* translated or purified from cell extract) \| IDA \| MI:0413 \| \|  \| nuclear extract from cells with ectopic expression of a mutated TF \| IMP \| MI:0413 \| \|  \| purified mutated TF (*in vitro* translated or purified from cell extract \| IMP \| MI:0413 \| \| Electrophoretic mobility supershift assay (EMSA supershift) \| nuclear extract from native tissue or cells \| IDA \| MI:0412 \| \|  \| nuclear extracts from cells or tissue with ectopic expression of a TF \| IDA \| MI:0412 \| \|  \| purified TF (*in vitro* translated or purified from cell extract) \| IDA \| MI:0412 \| \|  \| nuclear extract from cells with ectopic expression of a mutated TF \| IMP \| MI:0412 \| \|  \| purified mutated TF (*in vitro* translated or purified from cell extract) \| IMP \| MI:0412 \| \| Footprinting \|  \| IDA \| MI:0417 \| \| DNase I footprinting (DNA footprint) \|  \| IDA \| MI:0606 \| \| Methylation interference assay (MIC) \|  \| IDA \| MI:1189 \| \| Ultraviolet (UV) footprinting (UV-footprint) \|  \| IDA \| MI:1191 \| \| Dimethylsulphate footprinting (DMS-footprint) \|  \| IDA \| MI:0603 \| \| Hydroxy radical footprinting (Hydroxy-footprint) \|  \| IDA \| MI:1190 \| \| Potassium permanganate footprinting (KMnO4-footprint) \|  \| IDA \| MI:0604 \| \| Affinity chromatography technology \|  \| IDA \| MI:0004 \| \| Pull down \|  \| IDA \| MI:0096 \| \| Southwestern blot assay (SW-blot) \|  \| IDA \|  \| \| *In vitro* evolution of nucleic acids (SELEX) \|  \| IDA \| MI:0657 \| \| X-ray crystallography \|  \| IDA \| MI:0114 \| |  |
| --- | --- | --- | --- | --- | --- | --- | --- | --- | --- | --- | --- | --- | --- | --- | --- | --- | --- | --- | --- | --- | --- | --- | --- | --- | --- | --- | --- | --- | --- | --- | --- | --- | --- | --- | --- | --- | --- | --- | --- | --- | --- | --- | --- | --- | --- | --- | --- | --- | --- | --- | --- | --- | --- | --- | --- | --- | --- | --- | --- | --- | --- | --- | --- | --- | --- | --- | --- | --- | --- | --- | --- | --- | --- | --- | --- | --- | --- | --- | --- | --- | --- | --- | --- | --- | --- | --- | --- | --- | --- | --- | --- | --- | --- |

**Transcription regulation annotations**

| Use Table 2 to identify valid experimental evidence for one of the GO terms for transcription regulation shown in Figure 4.  Whenever possible, indicate whether the regulation is positive or negative, by using the adequate GO terms | 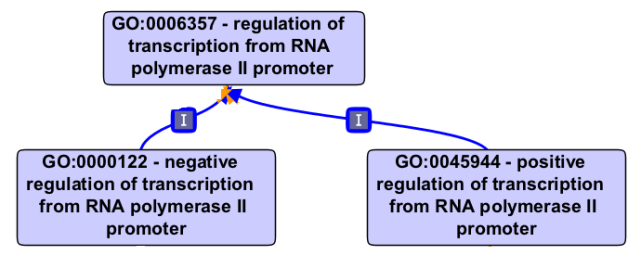  **Figure 4: GO terms for BP transcription regulation** | | |
| --- | --- | --- | --- |
| Transcription regulation annotations can also be supported by **whole organism experiments**, e.g. knock out mutations or RNAi knock down strategies.  Such experiments do not by themselves prove a role in regulation of transcription and must therefore be made with caution: they depend on a strict awareness of additional information such as the concomitant documentation of specific binding by the protein in question, to regulatory regions of an RNAP II regulated gene. | |  | |
|  |  | | |
| **Table 2: Reporter gene-based assays variants documenting transcription regulation.**   \| **TF identification** \| **Transcription regulation assays** \| \| \| \| \| \| \| \| --- \| --- \| --- \| --- \| --- \| --- \| --- \| --- \| \| ***reporter gene assay*** \| \| \| \| ***TG expression assay*** \| \| \| \| *canonical TFBS* \| *authentic TG promoter* \| *authentic TG promoter with TFBS point mutation* \| *authentic TG promoter with deletion mutations* \| *primer specific PCR*  *(e.g. RT-PCR, qRT-PCR)* \| *northern blot* \| *ribo-nuclease*  *protection assay* \| \| \| MI:0088 \| MI:0929 \| MI:0920 \| \| *wt TF overexpression* \| IDA \| IDA \| IDA \| IDA \| IDA \| IDA \| IDA \| \| *mut TF overexpression* \| IMP \| IMP \| IMP \| IMP \| IMP \| IMP \| IMP \| \| *TF knock down (RNAi/antisense RNA)* \| IMP \| IMP \| IMP \| IMP \| IMP \| IMP \| IMP \| | | |  |
|  | | |  |

**Sequence-specific DNA binding TF activity annotations**

| Based on the protein’s existing GO annotations for specific DNA-binding (MF) and for transcription regulation (BP), create a sequence-specific DNA-binding TF activity (MF) annotation.  Use Decision Table 3 to identify the correct GO term (shown in green in Figure 5). | 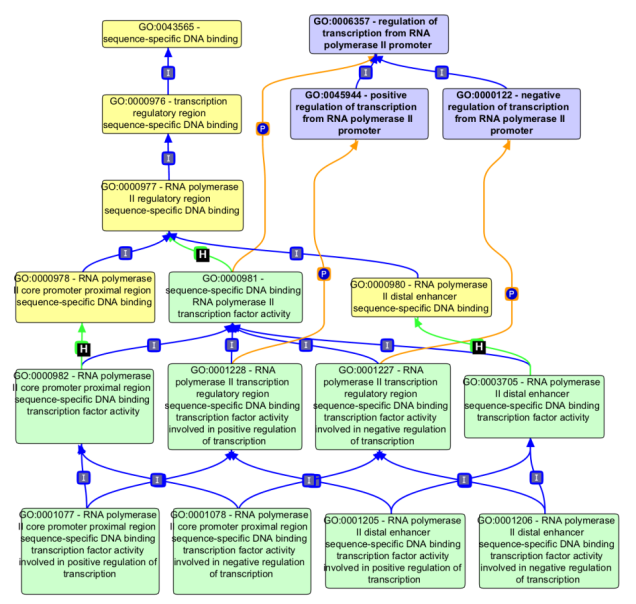  **Figure 5: GO terms for MF sequence-specific DNA binding TF activity** |
| --- | --- |
| Assign evidence codes according to Table 4 – see next page. |  |

| **Table 3: Inference of DbTF activity terms from sequence-specific DNA binding and transcription regulation terms**   \| ***Sequence-specific DNA binding terms (MF)*** \| ***Transcription regulation terms (BP)*** \| \| \| \| --- \| --- \| --- \| --- \| \| ***GO:0006357  regulation of transcription from RNA polymerase II promoter*** \| ***GO:0045944  positive regulation of transcription from RNA polymerase II promoter*** \| ***GO:0000122  negative regulation of transcription from RNA polymerase II promoter*** \| \| ***GO:0043565 sequence-specific DNA binding*** \| **GO: 0000981**  sequence-specific DNA binding RNA polymerase II transcription factor activity \| **GO:0001228**  RNA polymerase II transcription regulatory region sequence-specific DNA binding transcription factor activity involved in positive regulation of transcription \| **GO:0001227**  RNA polymerase II transcription regulatory region sequence-specific DNA binding transcription factor activity involved in negative regulation of transcription \| \| ***GO:0000976***  ***transcription regulatory region sequence-specific DNA binding*** \| \| ***GO:0000977  RNA polymerase II regulatory region sequence-specific DNA binding*** \| \| ***GO:0000978  RNA polymerase II core promoter proximal region sequence-specific DNA binding*** \| **GO:0000982**  RNA polymerase II core promoter proximal region sequence-specific DNA binding transcription factor activity \| **GO:0001077** RNA polymerase II core promoter proximal region sequence-specific DNA binding transcription factor activity involved in positive regulation of transcription \| **GO:0001078** RNA polymerase II core promoter proximal region sequence-specific DNA binding transcription factor activity involved in negative regulation of transcription \| \| ***GO:0000980  RNA polymerase II distal enhancer sequence-specific DNA binding*** \| **GO:0003705** sequence-specific distal enhancer binding RNA polymerase II transcription factor activity \| **GO:0001205**  RNA polymerase II distal enhancer sequence-specific DNA binding transcription factor activity involved in positive regulation of transcription \| **GO:0001206**  RNA polymerase II distal enhancer sequence-specific DNA binding transcription factor activity involved in negative regulation of transcription \| |  |
| --- | --- | --- | --- | --- | --- | --- | --- | --- | --- | --- | --- | --- | --- | --- | --- | --- | --- | --- | --- | --- | --- | --- |

**Evidence codes for sequence-specific DNA binding TF activity**

| Sequence-specific DNA binding (MF) and transcription regulation (BP) annotations from   - *same publication* and with *same evidence code* (either both IDA or both IMP), - sequence-specific DNA binding TF activity (MF) term receives this GO evidence code - *same publication* but with *different evidence codes* (IDA and IMP), - sequence-specific DNA binding TF activity (MF) term is repeated twice, once with each of the two GO evidence codes - *two different publications*: use GO evidence code ‘IC: Inferred by curator’.   **To generate GO evidence code ‘IC’:**  The two GO identifiers (specific DNA binding and transcription regulation) assigned to the same TF from two different publications are inserted into the 'with/from' field.  Reference GO_REF:0000036 is generated  (see also: http://www.geneontology.org/GO.evidence.shtml#ic)  **Table 4: Evidence code table**   \| **DNA binding** \| **Transcription regulation** \| **TF activity** \| \| --- \| --- \| --- \| \| IDA \| IDA \| IDA/IC \| \| IMP \| IMP \| IMP/IC \| \| IDA \| IMP \| IDA, IMP/IC \| \| IMP \| IDA \| IMP, IDA/IC \| |  |
| --- | --- | --- | --- | --- | --- | --- | --- | --- | --- | --- | --- | --- | --- | --- | --- | --- |

**TF binding TF activity annotations**

| Use Table 5 to identify valid experimental evidence for one of the GO terms for TF-binding shown in orange in Figure 6.  The IPI evidence code indicates that the interaction is a direct 1:1 interaction. The IDA evidence code should be used when the protein being annotated is shown to bind to a TF that is a complex.  The TF binding partner(s) must be recorded in ‘*with/from’* field (<http://www.geneontology.org/GO.evidence.shtml>).  Transcription regulation BP annotations (shown in blue in Figure 6) are made as described above for *Transcription Regulation annotations,* page 3.  Use Decision Table 6 to identify the correct GO term for TF-binding TF activity shown in pink in Figure 6.  For assignment of **evidence code for TF binding activity** see Table 7:  when TF binding (MF) and transcription regulation (BP) annotations are from   - *same publication* but with *different evidence codes* (IDA, IPI or IMP), - TF binding TF activity (MF) term is repeated twice or three times, once with each of the GO evidence codes - *two different publications*: use GO evidence code ‘IC: Inferred by curator’ (as described on page 5). | 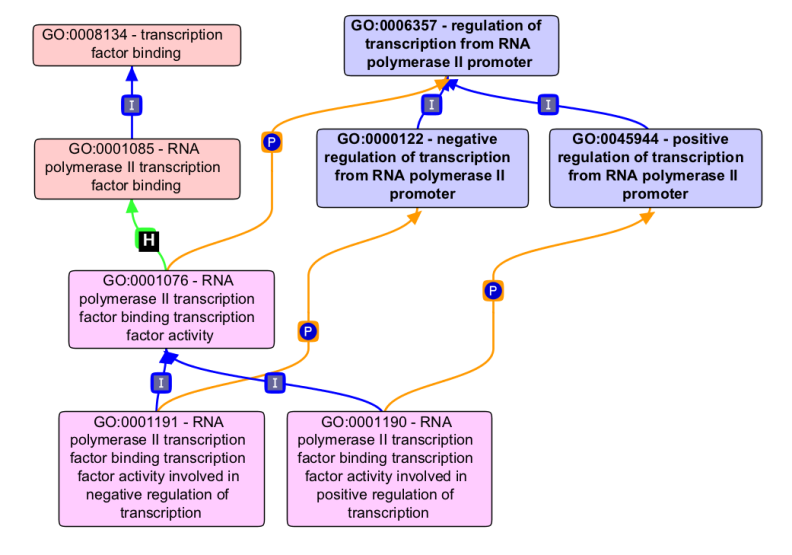  **Figure 6: GO terms for MF TF binding TF activity**  **Table 5: Assays documenting TF binding.**   \| **Assays** \| **GO evidence Code** \| **PSI-MI code** \| \| --- \| --- \| --- \| \| 2-hybrid interactions \| IPI \| MI:0018 \| \| Co-purification \| IPI, IDA \| MI:0004 \| \| Co-immunoprecipitation \| IPI, IDA \| MI:0019 \|   **Table 6: Inference of TF binding TF activity terms from TF binding and transcription regulation terms**  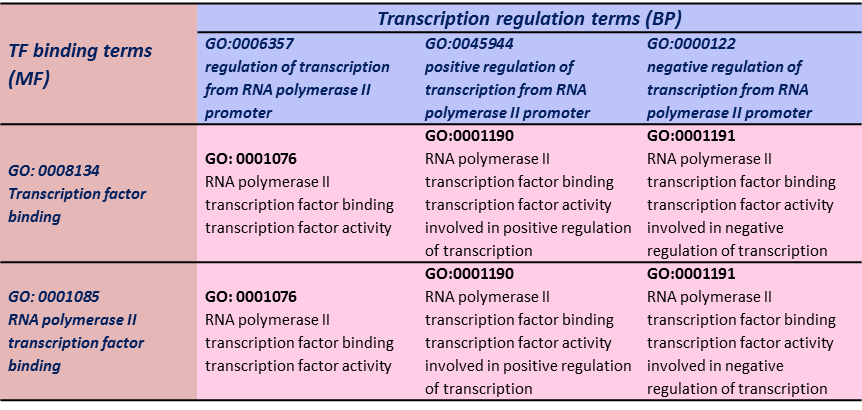  **Table 7: TF binding activity evidence code table**   \| **TF binding** \| **Transcription regulation** \| **TF activity** \| \| --- \| --- \| --- \| \| IDA \| IDA \| IDA/IC \| \| IMP \| IMP \| IMP/IC \| \| IDA \| IMP \| IDA, IMP/IC \| \| IMP \| IDA \| IMP, IDA/IC \| \| IPI \| IDA \| IPI, IDA/ IC \| |
| --- | --- | --- | --- | --- | --- | --- | --- | --- | --- | --- | --- | --- | --- | --- | --- | --- | --- | --- | --- | --- | --- | --- | --- | --- | --- | --- | --- | --- | --- | --- | --- |

**When the functional unit of a TF is a complex**

| When the complex is a **homodimer, or higher order multimer of the same protein**, there are no special annotation issues as all of the activities demonstrated are properties of the same gene product.  **Additional considerations for heterodimers and other multisubunit complexes**  The **“contributes to” qualifier** must always be used to indicate the annotation of molecular functions that occur in the context of complexes. The “contributes to” qualifier can be used in conjunction with any MF term, including the “transcription factor activity” terms, to indicate that it contributes to that function within the context of a complex, even though it does not possess that activity independently  In the case of a **heterodimer** **where one of the two proteins does not bind DNA on its own** **but is still found to contribute to the sequence specific binding of the other subunit** within a heterodimer: the subunit that does not bind DNA alone can still be annotated to “sequence-specific DNA binding”, or possibly a more specific term, using the qualifier “contributes to” to indicate that it contributes to the DNA binding of the heterodimer.  In a **multisubunit TF where the DNA binding activity is known to be confined to one or more specific subunits**: other subunits should **not** be annotated to a “DNA binding” term.  **For any subunit within a TF complex**, it is appropriate to annotate to all appropriate GO terms for which that function has been experimentally shown, either individually or as part of the complex indicated with the “contributes to” qualifier. Thus, in some cases, a given protein may be annotated both a ‘sequence specific DNA binding RNAP II transcription factor activity’ term as well as a ‘TF binding RNAP II transcription factor activity’ term. |  |
| --- | --- |
|  |  |

**Target gene (TG) annotations**

Use the ‘annotation extension’ column shown in Figure 7 to capture information for one or several TGs shown to be regulated by the TF whose function is annotated by using “has_regulation_target”, source of TG identifier and TG identifier, as explained below.


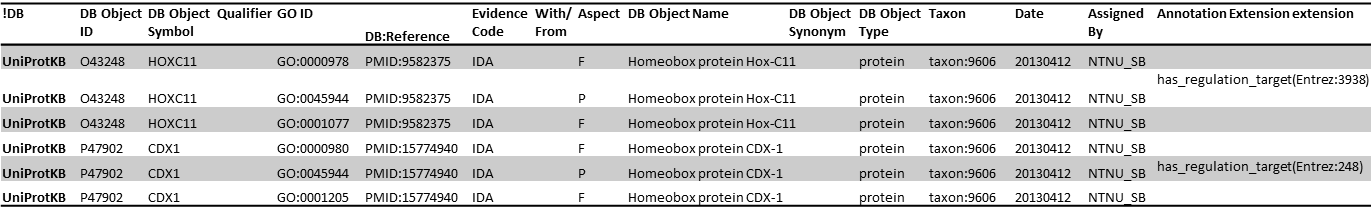


**Figure 7:** DbTF sample annotations with TG information (rows 2 and 5) shown as “column 16” of the spread sheet GAF2.0 format; http://www.geneontology.org/GO.format.gaf-2_0.shtml.

The relationship ‘**’has_regulation_target’’** is used to capture TG information in the spread sheet row used to record the transcription regulation term. This row can either hold terms that have is_a relationships to the term “biological regulation” (i.e. are BP-terms), or MF terms representing regulators that are part of regulatory processes (i.e. have part_of relationships to a BP regulation term).

In the examples shown in Figure 7, the transcriptiojn regulation term is GO:0045944 *positive regulation of transcription from RNA polymerase II promoter*.

When we use the “has_regulation_target” relationship, we are saying that the GO term used for the annotation, e.g. “*regulation of transcription from RNA polymerase II*” or “*sequence-specific DNA binding RNA polymerase II transcription factor activity*” has a target, and we use a gene ID (URI) to specify what that target is.

To indicate multiple TGs in the same annotation: separate each 'relationship(identifier)' pair with a pipe, “|”.

**Example:** to capture the two TGs, the annotation extension column should contain:

has_regulation_target(source:GeneURI1) | has_regulation_target (source:GeneURI2)

where **source** can be ENSEMBL, Entrez, or a model organism database, e.g. MGI, RGD, etc. and **Gene URI1** and **GeneURI2** denote identifiers from any of the above sources.

**GO terms**

**Transcription regulation terms**

[**GO:0006357 regulation of transcription from RNA polymerase II promoter**](http://amigo.geneontology.org/cgi-bin/amigo/term_details?term=GO:0006357#lineage)

**Definition:** “Any process that modulates the frequency, rate or extent of transcription from an RNA polymerase II promoter.”

[**GO:0045944 positive regulation of transcription from RNA polymerase II promoter**](http://amigo.geneontology.org/cgi-bin/amigo/term_details?term=GO:0045944#lineage)

**Definition:** “Any process that activates or increases the frequency, rate or extent of transcription from an RNA polymerase II promoter.”

[**GO:0000122 negative regulation of transcription from RNA polymerase II promoter**](http://amigo.geneontology.org/cgi-bin/amigo/term_details?term=GO:0000122#lineage)

**Definition:** “Any process that stops, prevents, or reduces the frequency, rate or extent of transcription from an RNA polymerase II promoter.”

**Sequence-specific DNA binding terms**

**GO:0043565 - sequence-specific DNA binding**

**Definition:** "Interacting selectively and non-covalently with DNA of a specific nucleotide composition, e.g. GC-rich DNA binding, or with a specific sequence motif or type of DNA e.g. promotor binding or rDNA binding."

**GO:0000976 - transcription regulatory region sequence-specific DNA binding**

**Definition:** Interacting selectively and non-covalently with a specific sequence of DNA that is part of a regulatory region that controls transcription of that section of the DNA. The transcribed region might be described as a gene, cistron, or operon.

**GO:0000977 - RNA polymerase II regulatory region sequence-specific DNA binding**

**Definition:** "Interacting selectively and non-covalently with a specific sequence of DNA that is part of a regulatory region that controls the transcription of a gene or cistron by RNA polymerase II."

**GO:0000978 - RNA polymerase II core promoter proximal region sequence-specific DNA binding**

**Definition:** "Interacting selectively and non-covalently with a sequence of DNA that is in cis with and relatively close to a core promoter for RNA polymerase II."

comment: Note that the phrase "upstream activating sequence", or UAS is often used in S. cerevisiae literature to refer to regulatory sequences that occur in the region upstream and proximal to the core promoter. In contrast, in bacteria such as E. coli, the phrase "upstream activating sequence", or UAS is a synonym for "enhancer".

**GO:0000980 - RNA polymerase II distal enhancer sequence-specific DNA binding**

**Definition:** "Interacting selectively and non-covalently with a RNA polymerase II (Pol II) distal enhancer. In mammalian cells, enhancers are distal sequences that increase the utilization of some promoters, and can function in either orientation and in any location (upstream or downstream) relative to the core promoter."

**Sequence-specific DNA binding transcription factor activity terms**

**GO: 0000981 - sequence-specific DNA binding RNA polymerase II transcription factor activity**

**Definition:** Interacting selectively and non-covalently with a specific DNA sequence in order to modulate transcription by RNA polymerase II. The transcription factor may or may not also interact selectively with a protein or macromolecular complex.

**GO:0001227 - RNA polymerase II transcription regulatory region sequence-specific DNA binding transcription factor activity involved in negative regulation of transcription**

**Definition:** Interacting selectively and non-covalently with a sequence of DNA that is in the regulatory region for RNA polymerase II (RNAP II) in order to stop, prevent, or reduce the frequency, rate or extent of transcription from an RNA polymerase II promoter.

**GO:0001228 - RNA polymerase II transcription regulatory region sequence-specific DNA binding transcription factor activity involved in positive regulation of transcription**

**Definition:** Interacting selectively and non-covalently with a sequence of DNA that is in the transcription regulatory region for RNA polymerase II (RNAP II) in order to activate or increase the frequency, rate or extent of transcription from the RNAP II promoter.

**GO:0000982 - RNA polymerase II core promoter proximal region sequence-specific DNA binding transcription factor activity**

**Definition:** Interacting selectively and non-covalently with a sequence of DNA that is in cis with and relatively close to a core promoter for RNA polymerase II (RNAP II) in order to modulate transcription by RNAP II.

**GO:0001077- RNA polymerase II core promoter proximal region sequence-specific DNA binding transcription factor activity involved in positive regulation of transcription**

**Definition :** Interacting selectively and non-covalently with a sequence of DNA that is in cis with and relatively close to a core promoter for RNA polymerase II (RNAP II) in order to activate or increase the frequency, rate or extent of transcription from the RNAP II promoter.

**GO:0001078- RNA polymerase II core promoter proximal region sequence-specific DNA binding transcription factor activity involved in negative regulation of transcription**

**Definition:** Interacting selectively and non-covalently with a sequence of DNA that is in cis with and relatively close to a core promoter for RNA polymerase II (RNAP II) in order to stop, prevent, or reduce the frequency, rate or extent of transcription from the RNAP II promoter.

**GO:0003705 - sequence-specific distal enhancer binding RNA polymerase II transcription factor activity**

**Definition:** Interacting selectively and non-covalently with a sequence of DNA that is in a distal enhancer region for RNA polymerase II (RNAP II) in order to modulate transcription by RNAP II.

**GO:0001205** - **RNA polymerase II distal enhancer sequence-specific DNA binding transcription factor activity involved in positive regulation of transcription**.

**Definition:** “Interacting selectively and non-covalently with a sequence of DNA that is in a distal enhancer region for RNA polymerase II (RNAP II) in order to activate or increase the frequency, rate or extent of transcription from the RNAP II promoter.”

**GO:0001206** - **RNA polymerase II distal enhancer sequence-specific DNA binding transcription factor activity involved in negative regulation of transcription**.

**Definition:** “Interacting selectively and non-covalently with a sequence of DNA that is in a distal enhancer region for RNA polymerase II (RNAP II) in order to stop, prevent, or reduce the frequency, rate or extent of transcription from an RNA polymerase II promoter.”

**Transcription factor binding terms**

**GO:0008134 - Transcription factor binding (MF)**

**Definition:** Interacting selectively and non-covalently with a transcription factor, any protein required to initiate or regulate transcription.

**GO:0001085 - RNA polymerase II transcription factor binding (MF)**

**Definition:** Interacting selectively and non-covalently with an RNA polymerase II transcription factor, any protein required to initiate or regulate transcription by RNA polymerase II.

**Transcription factor binding transcription factor activity terms**

**GO:0001076 - RNA polymerase II transcription factor binding transcription factor activity (BP)**

**Definition:** Interacting selectively and non-covalently with an RNA polymerase II transcription factor, which may be a single protein or a complex, in order to modulate transcription. A protein binding transcription factor may or may not also interact with the template nucleic acid (either DNA or RNA) as well.

**GO:0001190 - RNA polymerase II transcription factor binding transcription factor activity involved in positive regulation of transcription (BP)**

**Definition:** Interacting selectively and non-covalently with an RNA polymerase II transcription factor, which may be a single protein or a complex, in order to increase the frequency, rate or extent of transcription from an RNA polymerase II promoter. A protein binding transcription factor may or may not also interact with the template nucleic acid (either DNA or RNA) as well.

**GO:0001191 - RNA polymerase II transcription factor binding transcription factor activity involved in negative regulation of transcription (BP)**

**Definition:** Interacting selectively and non-covalently with an RNA polymerase II transcription factor, which may be a single protein or a complex, in order to stop, prevent, or reduce the frequency, rate or extent of transcription from an RNA polymerase II promoter. A protein binding transcription factor may or may not also interact with the template nucleic acid (either DNA or RNA) as well.

**GOC evidence codes**

**IDA – Inferred from direct assay**

**Description (GOC):** The IDA evidence code is used to indicate that a direct assay was carried out to determine the function, process, or component indicated by the GO term.

**IMP – Inferred from mutant phenotype**

**Description (GOC):** The IMP evidence code covers those cases when the function, process or cellular localization of a gene product is inferred based on differences in the function, process, or cellular localization between two different alleles of the corresponding gene. The IMP code is used for cases where one allele may be designated 'wild-type' and another as 'mutant'. It is also used in cases where allelic variation occurs naturally and no specific allele is designated as wild-type or mutant.

**IC - Inferred by Curator**

**Description (GOC):** The IC evidence code is to be used for those cases where an annotation is not supported by any direct evidence, but can be reasonably inferred by a curator from other GO annotations, for which evidence is available.

**IPI - Inferred from Physical Interaction**

**Description (GOC):** Covers physical interactions between the gene product of interest and another molecule (such as a protein, ion or complex). IPI can be thought of as a type of IDA, where the actual binding partner or target can be specified, using "with" in the with/from field.
